# Clinical Characterization of the Lacrimal Functional Unit in Patients with Chronic Ocular Pain Associated with Dry Eye Disease

**DOI:** 10.3390/jcm14155250

**Published:** 2025-07-24

**Authors:** Marta Blanco-Vázquez, Andrea Novo-Diez, Amanda Vázquez, Amalia Enríquez-de-Salamanca, María J. González-García, Margarita Calonge

**Affiliations:** 1 Institute of Applied Ophthalmobiology (IOBA), Universidad de Valladolid (UVa), Campus Miguel Delibes, Paseo de Belén 17, 47011 Valladolid, Spain; marta.blanco.vazquez@uva.es (M.B.-V.); andrea.novo@uva.es (A.N.-D.); avazquezhs@gmail.com (A.V.); amalia.enriquez-salamanca@uva.es (A.E.-d.-S.); mcalonge@uva.es (M.C.); 2 Biomedical Research Networking Center in Bioengineering, Biomaterials and Nanomedicine (CIBER-BBN), Campus Miguel Delibes, Paseo de Belén 17, 47011 Valladolid, Spain

**Keywords:** chronic ocular pain, dry eye disease, lacrimal functional unit, in vivo confocal microscopy

## Abstract

**Background/Objectives**: The purpose of this study was to clinically characterize the lacrimal functional unit (LFU) of patients with chronic ocular pain associated with dry eye disease (DED). **Methods**: Ninety-three participants were included in this cross-sectional study: 28 patients with chronic ocular pain associated with DED (pain-DED), 35 patients with DED but no pain (no pain-DED), and 30 subjects without DED or ocular pain (controls). The following examinations were performed: symptom questionnaires, visual function assessment, tear meniscus, ocular surface evaluation, meibography, corneal sensitivity, Schirmer test, and in vivo corneal confocal microscopy. **Results**: Both DED groups presented increased DED-related symptoms (*p* < 0.001), corneal staining (*p* < 0.001), Meibomian gland loss (*p* < 0.010), and dendritic cell density (*p* < 0.001) compared with controls. Comparing both DED groups, the pain-DED group showed higher DED-related symptoms (*p* < 0.002) and increased microneuroma density (*p* < 0.001). Additionally, significant positive correlations were observed between symptom questionnaires and corneal staining (vs. OSDI: r = 0.514, *p* < 0.001; vs. m-SIDEQ: r = 0.504, *p* < 0.001; vs. NRS: r = 0.361, *p* < 0.001; vs. WBFPRS: r = 0.317, *p* = 0.002), dendritic cell density (vs. OSDI: r = 0.429, *p* < 0.001; vs. m-SIDEQ: r = 0.440, *p* < 0.001), and microneuroma density (vs. NRS: r = 0.405, *p* < 0.001; vs. WBFPRS: r = 0.416, *p* < 0.001). **Conclusions**: Differences in the LFU, especially in the morphology of sub-basal corneal nerves, are related to the presence of DED and chronic ocular pain and, along with ocular clinical questionnaires, can help phenotype these patients.

## 1. Introduction

DED is an inflammatory disorder of the Lacrimal Functional Unit (LFU) [1] that can be initiated by multiple causes, leading to a wide range of symptoms [2], including ocular pain [3], which can become chronic if it persists for longer than 3 months [4]. Chronic pain is recognized as a disease in its own right [5] and represents a global public health concern [6], with a reported worldwide prevalence of 38.4% [7]. Chronic ocular pain negatively affects patients’ quality of life, similarly to DED, by interfering with daily activities, contributing to mental health disorders, and even resulting in occupational disability, impaired social functioning, and suicidal ideation [6,8,9].

The cornea stands out as the most densely innervated tissue in the human body. It is mainly innervated by the ophthalmic branch of the trigeminal nerve (cranial nerve V), and most corneal nerve fibers are sensory in origin [10]. Free corneal nerve endings are located between superficial epithelium cells and are thus in close contact with the environment, which makes them vulnerable to repeated damage [10,11]. Several stimuli, including mechanical, chemical, and thermal insults, as well as adverse environmental conditions, inflammation, infection, trauma, abnormal ocular anatomy, and tear hyperosmolarity, can trigger ocular surface damage [11]. This damage to epithelial cells and corneal nerves can lead to the release of inflammatory mediators, which in turn can alter the activity of nociceptors and elicit pain sensations [11]. However, the mechanisms underlying this ocular pain are not fully understood, and further research needs to be conducted [12,13]. Additionally, ocular pain can be initiated in other parts of the globe different from the cornea, and thus, this is why the term ocular pain is more correct and inclusive than corneal pain.

Diagnosing ocular pain remains challenging due to the lack of standardized clinical criteria [14]. Current approaches rely primarily on the use of subjective symptom questionnaires and pain-rating scales. However, the absence of a diagnostic “gold standard” has led to a growing interest in further characterizing ocular pain associated with DED and in identifying objective tests that could support diagnosis and help management [3]. In this regard, the assessment of corneal nerve morphology using in vivo confocal microscopy and the evaluation of corneal sensitivity by esthesiometry could contribute to a better diagnosis and management by providing additional insight into the mechanisms underlying ocular pain [3].

Thus, the present study has focused on evaluating the clinical characteristics of the LFU of patients with chronic ocular pain associated with DED and comparing them with those of patients with DED but no pain and healthy controls. These findings will contribute to a deeper understanding of the underlying mechanisms of this condition. In addition, they may serve as tools for the diagnosis and monitoring of these patients and support the development of effective and personalized therapies.

## 2. Materials and Methods

This cross-sectional, case–control study was conducted at a single center and received approval from the Ethics Committee of “Area de Salud Valladolid-Este” (Valladolid, Spain) (Reference number: PI 15-301). All procedures followed the principles outlined in the Declaration of Helsinki and adhered to Good Clinical Practice guidelines. Participants were informed about the purpose and nature of the study, and written consent was obtained before their participation.

### 2.1. Patient Sample

Three groups of individuals were included: (1) patients with chronic ocular pain associated with DED (pain-DED); (2) patients with DED but no pain (no pain-DED); and (3) subjects without DED or ocular pain (controls).

The diagnosis of DED was established based on an Ocular Surface Disease Index (OSDI) score ≥ 13, along with the presence of at least two of the following four signs in both eyes: (1) tear break-up time (TBUT) ≤ 7 s; (2) corneal fluorescein staining ≥ grade 1 (Oxford grading scheme); (3) conjunctival lissamine green staining ≥ grade 1 (Oxford grading scheme); and (4) Schirmer test with topical anesthesia ≤ 5 mm in 5 min [15].

Ocular pain was considered as present when both the Numerical Rating Scale (NRS) and the Wong–Baker Faces^®^ Pain-Rating Scale (WBFPRS) yielded scores of ≥2 [15,16]. Pain was considered chronic if it had been present for a minimum of 3 months [17].

Inclusion criteria included the following [15]: (1) individuals aged 18 years or older and (2) diagnosis of DED and chronic ocular pain in the respective groups, as per the above definitions. Participants were excluded if they met any of the following conditions: (1) presence of ocular surface disease within the past 3 months or ocular surgery within the previous 6 months (excluding DED and chronic ocular pain in the respective groups); (2) diagnosis of a systemic/extraocular disease with potential ocular involvement (including ocular pain) in the past 3 months; (3) initiation of systemic treatment potentially affecting the LFU in the 3 months prior to enrollment; (4) commencement of lacrimal drainage system occlusion within the last 3 months; (5) use of contact lenses within 7 days before the enrollment; (6) use of any topical ocular medication (including cyclosporine A and corticosteroids) in the month preceding the study; (7) use of topical ocular lubricants or blood derivatives within 12 h prior to the study.

Controls were required to fulfill the same inclusion and exclusion criteria, except for the inclusion diagnostic criteria of DED and chronic ocular pain.

### 2.2. Study Design

A single visit was conducted for this study. Prior to clinical evaluation, all participants underwent a 30 min adaptation period under normal controlled environmental conditions (23 °C, 50% relative humidity, and absence of localized air flow) within the exposure room of the Controlled Environment Laboratory (CELab) (www.visionrd.com/celab/, accessed on 14 May 2019) [18]. The purpose of this was to normalize the environmental conditions in which clinical evaluations were conducted and minimize the effects of a changing external environment [19]. During this period, participants’ medical histories were recorded, and symptom-related questionnaires were administered. Subsequently, the clinical examination was also conducted under normal controlled conditions, always between 9:30 am and 2:00 pm.

### 2.3. Symptoms

DED-related symptoms were evaluated using the OSDI questionnaire, and the modified Single-Item Score Dry Eye Questionnaire (m-SIDEQ), while ocular pain intensity was assessed with the NRS and the WBFPRS. In addition, anxiety and depression states were evaluated using the Hospital Anxiety and Depression Scale (HADS).

The OSDI is a 12-item questionnaire that evaluates the frequency of DED-related symptoms, the limitations in performing visual activities, and the sensation of discomfort under dry environmental conditions. This questionnaire ranges from 0 to 100 and classifies DED subjects as follows: 0–12 = absence of symptoms; 13–22 = mild DED-symptoms; 23–32 = moderate DED-symptoms; and 33–100 = severe DED-symptoms [20].

The m-SIDEQ is a 7-item questionnaire that assesses the symptoms of dryness, sandy or gritty feeling, burning or stinging, pain, itching, sensitivity to light, and blurred vision. Each symptom is scored on a 5-point scale (0–4 scale, where 0 = absence of symptom; 1 = seldom felt, but does not cause discomfort or interfere with activities; 2 = sometimes felt, causes discomfort but does not interfere with activities; 3 = frequently felt, causes discomfort, and sometimes interferes with activities; and 4 = always felt, causes discomfort, and usually interferes with activities), and the sum of the 7 items is scored from 0 to 28 [21].

The NRS is an 11-point scale widely used to measure pain intensity. It consists of a line numbered from 0 to 10, where 0 represents no pain and 10 the worst possible pain. Participants selected the number that best represented their ocular pain intensity [22].

The WBFPRS is a 6-point scale commonly used to measure pain intensity in children and adults. It consists of six different faces representing increasing levels of pain intensity. Each face corresponds to a numeric score. Participants selected the face that best represented their ocular pain intensity [16].

The HADS is a 14-item questionnaire that evaluates the states of anxiety and depression and measures their severity. It consists of two 7-item subscales (anxiety subscale and depression subscale). Each item is scored on a 4-point scale (0–3 scale), and the sum of the 7 items is scored from 0 to 21 for anxiety and depression, respectively. Depending on the score obtained in each subscale, anxiety and depression states are classified as 0–7 = normal, no presence of anxiety and/or depression; 8–10 = possible presence of anxiety and/or depression; and 11–21 = probable presence of anxiety and/or depression [23].

### 2.4. Clinical Tests

Monocular visual acuity with habitual correction was measured using high- (100%) and low-contrast (10%) Early Treatment Diabetic Retinopathy Study (ETDRS) charts (CC-100 LCD system; Topcon Corporation, Tokyo, Japan) at a 4 m distance. Results were recorded in LogMAR units (−0.02 per correctly identified letter).

Higher-order aberrations were measured using the IRX3 aberrometer (Imagine Eyes, Orsay, France) under scotopic light conditions. The pupil diameter was set at 3 mm to include the natural pupil diameter of all participants. The root mean square of the total higher-order aberrations from third to sixth order was recorded.

Lower tear meniscus images were captured using the Topcon 3D OCT-2000 (Topcon Corporation). A vertical cross-sectional scan centered on the inferior cornea and the lower eyelid was taken using a 6 mm vertical line anterior segment tool. Then, ImageJ software version 1.52s (https://imagej.nih.gov/ij/, accessed on 6 April 2020) was used to measure the tear meniscus height, depth, area, and angle [24,25].

The lipid layer pattern was evaluated with the EasyTear^®^ View+ interferometer (EASYTEAR s.r.l., Rovereto, Italy). Then, the interference pattern was evaluated using a 5-point grading scale described by Yokoi N et al. [26].

Ocular surface examination was conducted using a SL-D7 slit lamp (Topcon Corporation), and the following parameters were evaluated: conjunctival hyperemia, TBUT, corneal and conjunctival staining, lid wiper epitheliopathy, lid margin, and meibum secretion. Bulbar conjunctival hyperemia was evaluated using the Efron grading scale (0–4 scale) [27]. Tarsal conjunctival hyperemia was graded using the Cornea and Contact Lens Research Unit (CCLRU) (0–4 scale) [28]. Nasal and temporal lid parallel conjunctival folds were counted. Three consecutive TBUT measurements were obtained using sodium fluorescein strips (I-DEW FLO, Entod Research Cell UK Ltd., London, UK) wetted with sodium chloride solution, and the average value was recorded. The extent of corneal staining was evaluated with two grading scales: the 0–20 CCLRU scale [28] and the 0–5 Oxford scale [29]. Conjunctival staining extent was assessed according to the Oxford grading scale (0–5 scale) [29] using lissamine green strips (I-DEW green, Entod Research Cell UK Ltd.) wetted with sodium chloride solution. It was analyzed in nasal and temporal areas, and then the average was calculated and considered as the total conjunctival staining. Lid wiper epitheliopathy was examined using the grading-score system of Korb et al. [30]. Blepharitis was graded using the Efron grading scale (0–4 scale) [27]. The quality and expressibility of meibum secretion were assessed by applying digital pressure on the upper and lower eyelids and graded using the Bron et al. scale (0–3 scale) [31] and the Shimazaki et al. scale (0–3 scale) [32], respectively. Also, the number of obstructed Meibomian glands was recorded.

Images of the Meibomian gland morphology were captured with the EasyTear^®^ View+ system and the MV500 USB Digital Microscope accessory (EASYTEAR s.r.l.). Then, the percentage of Meibomian gland loss was evaluated in each eyelid using a 5-grade meiboscale [33].

Corneal sensitivity was measured at the corneal apex using two esthesiometers: Belmonte non-contact esthesiometer and Cochet–Bonnet contact esthesiometer (Luneau Ophthalmology, Chartres, France). Mechanical and thermal (cold and heat) thresholds were determined by the method of levels using the non-contact esthesiometer [34,35,36,37]. Corneal tactile sensitivity was assessed using the Cochet–Bonnet esthesiometer before and after topical anesthesia [38].

The anesthetic challenge test was performed before the second corneal tactile sensitivity threshold measured with the Cochet–Bonnet esthesiometer. One minute after topical anesthesia, participants rated the change in intensity of their current ocular symptoms post-anesthesia using the Global Rating of Change (GRC) scale [39].

Basal tear production was assessed via the Schirmer test with topical anesthesia using sterile Schirmer strips (I-DEW tear strips, Entod Research Cell UK Ltd.).

In vivo confocal microscopy was conducted under topical anesthesia with the Heidelberg Retina Tomograph III and the Rostock Cornea Module (Heidelberg Engineering GmbH, Heidelberg, Germany). A disposable TomoCap (Heidelberg Engineering GmbH) with a drop of Viscotears gel (Alcon, Fort Worth, TX, USA) applied to its inner surface was placed on the microscope objective lens. Then, a drop of Viscotears gel was applied to the outer contact surface of the cap to provide the corneal epithelium with lubrication and protection from friction during movement. Participants were asked to stare at a fixation point situated straight ahead, and the tip of the TomoCap was placed in contact with the central cornea. Images from the central area of the sub-basal corneal nerve plexus were then captured using sequence scans. Each image has a resolution of 384 × 384 pixels, which represents a coronal section of 400 × 400 μm (0.16 mm^2^). Three good-quality, non-overlapped images were analyzed using the ImageJ software and its plugin NeuronJ version 1.4.3 (https://imagescience.org/meijering/software/neuronj/, accessed on 5 May 2020). Two mask observers measured the following parameters: (1) total nerve number, (2) total nerve density (evaluated as the total nerve length per frame), (3) mean nerve length, (4) nerve tortuosity according to the 0–4 scale reported by Oliveira-Soto and Efron [40], (5) image reflectivity (measured as an index of mean plexus reflectivity or optic densitometry using ImageJ’s histogram function), (6) density of nerve branch points (evaluated as nerve bifurcations per frame), (7) dendritic cell density, and (8) microneuroma density.

### 2.5. Statistical Analysis

The sample size was estimated using G*Power software (version 3.1.9.4) [41] to detect clinical differences via a one-way analysis of variance (ANOVA) with a three-level factor.

A statistical power of 80% and a level of significance of 1.67% (adjusted to control for experiment-wise type I error across multiple comparisons) were set. To detect a large effect size (Cohen’s Coefficient, f, of 0.40) as statistically significant, a minimum of 28 participants per group was required [42].

The SPSS Statistics 24 for Windows (IBM Corp., Armonk, NY, USA) was used to perform statistical analysis of the data [43]. Comparisons among groups as well as correlations were carried out. Clinical examination was conducted on both eyes, but one eye was randomly selected for statistical analysis. Quantitative variables are expressed as mean value and standard deviation; nominal variables are reported as frequencies; ordinal variables are presented as median and interquartile range. In the figures, data are presented as median and interquartile range, with the mean represented by a cross.

Quantitative variables were tested for normality using the Kolmogorov–Smirnov test and for homogeneity of variances using the Levene test. For comparisons among the three groups, the one-way ANOVA was applied to normally distributed variables, while the Kruskal–Wallis H test was used for non-normally distributed data. Nominal variables were analyzed using the Chi-square test, and ordinal variables were examined using the Kruskal–Wallis H test. Subsequently, post hoc pairwise comparisons of normally distributed quantitative variables were analyzed via the Tukey test, whereas the Mann–Whitney U test and the Bonferroni correction were used for non-normally distributed quantitative variables and ordinal variables. Additionally, for confocal microscopy parameters, the intraclass correlation coefficient (ICC) was calculated to assess the agreement between the two observers.

In addition, correlations between quantitative parameters were analyzed in the whole sample. For variables with a normal distribution, the Pearson correlation coefficient was applied, whereas the Spearman correlation coefficient was used for non-normally distributed variables. A *p*-value ≤ 0.05 was considered statistically significant.

## 3. Results

### 3.1. Sample Description

The study included 93 subjects distributed among the three groups as follows: 28 (30.1%) pain-DED, 35 (37.6%) no pain-DED, and 30 (32.3%) controls. A total of four participants were diagnosed with Sjögren’s syndrome, representing 7.14% (2 participants) of the pain-DED group and 5.71% (2 participants) of the no pain-DED group.

The demographic characteristics of the three groups are presented in Table 1.

### 3.2. Symptoms

**Figure 1:**
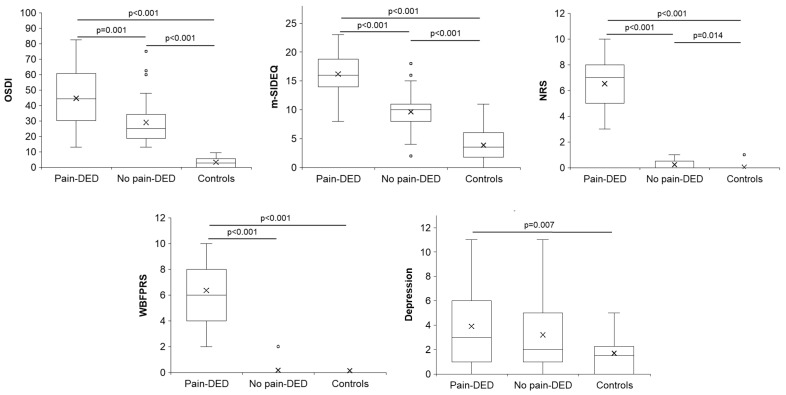
Post hoc pairwise comparisons of the symptom questionnaires with significant differences. DED: dry eye disease; m-SIDEQ: modified Single-Item score Dry Eye Questionnaire; NRS: Numerical Rating Scale; OSDI: Ocular Surface Disease Index; WBFPRS: Wong–Baker Faces^®^ Pain-Rating Scale.

The comparison among the three groups regarding symptom questionnaires is presented in Table S1 (Supplementary S1). Statistically significant differences were observed in the severity and frequency of DED-related symptoms (OSDI and m-SIDEQ, respectively), both ocular pain intensity scales (NRS and WBFPRS), and depression. Significant post hoc pairwise comparisons are shown in Figure 1.

### 3.3. Clinical Tests

The comparison among the three groups regarding visual acuity and higher-order aberrations is presented in Table S2 (Supplementary S1). No statistically significant differences were found.

**Figure 2:**
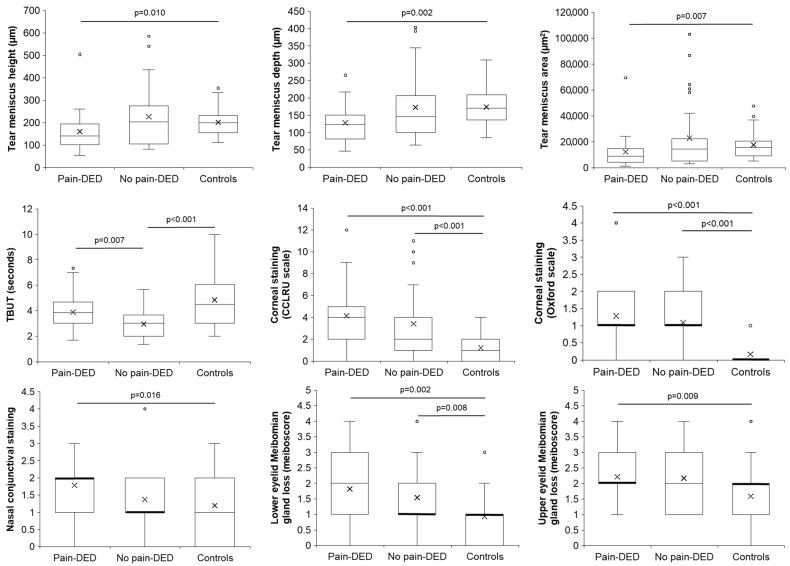
Post hoc pairwise comparisons of the slit-lamp findings with significant differences. CCLRU: Cornea and Contact Lens Research Unit; DED: dry eye disease; TBUT: tear break-up time.

The comparison among the three groups regarding the tear meniscus parameters, lipid layer pattern, slit-lamp findings, Meibomian gland loss, and Schirmer test is shown in Table S3 (Supplementary S1). Statistically significant differences were observed in the tear meniscus height, depth and area, TBUT, corneal and nasal conjunctival staining, meibum secretion expressibility, and Meibomian gland loss of both eyelids. Significant post hoc pairwise comparisons are shown in Figure 2.

**Figure 3:**
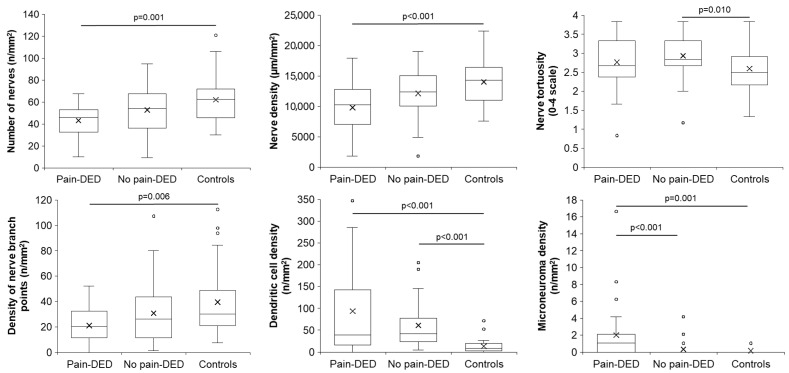
Post hoc pairwise comparisons of the in vivo confocal microscopy parameters with significant differences. DED: dry eye disease.

The comparison among the three groups regarding corneal sensitivity and in vivo confocal microscopy parameters is presented in Table S4 (Supplementary S1). Statistically significant differences were observed in the number of nerves, nerve density, nerve tortuosity, density of nerve branch points, dendritic cell density, and microneuroma density. Significant post hoc pairwise comparisons are shown in Figure 3.

Additionally, regarding the parameters of sub-basal corneal nerve plexus, the agreement between the two observers was high for the evaluation of the number of nerves (ICC = 0.94, 95% confidence interval: 0.91, 0.96), nerve density (ICC = 0.97, 95% confidence interval: 0.89, 0.99), nerve length (ICC = 0.82, 95% confidence interval: 0.43, 0.92), image reflectivity (ICC = 1.00, 95% confidence interval: 1.00, 1.00), density of nerve branch points (ICC = 0.94, 95% confidence interval: 0.90, 0.96), dendritic cell density (ICC = 0.96, 95% confidence interval: 0.94, 0.97), and microneuroma density (ICC = 0.95, 95% confidence interval: 0.92, 0.97). However, it was moderate for the assessment of the grade of nerve tortuosity (ICC = 0.61, 95% confidence interval: 0.29, 0.77).

### 3.4. Correlations Among Clinical Parameters

**Figure 4:**
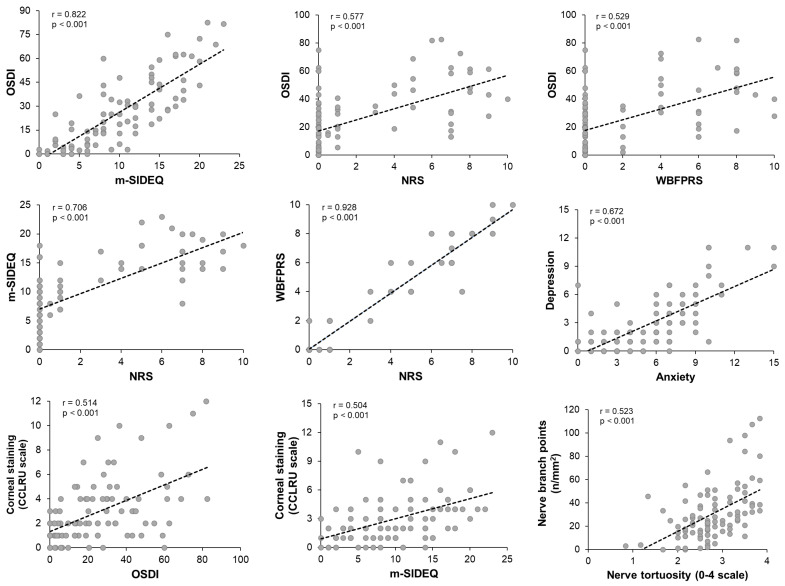
Significant and relevant correlations with correlation coefficients higher than 0.5. m-SIDEQ: modified Single-Item score Dry Eye Questionnaire; NRS: Numerical Rating Scale; OSDI: Ocular Surface Disease Index; WBFPRS: Wong–Baker Faces^®^ Pain-Rating Scale.

All correlations among the studied parameters are included in Tables S5–S9 (Supplementary S2). Significant and clinically relevant correlations with correlation coefficients higher than 0.5 are graphically represented in Figure 4.

Additionally, numerous moderate correlations of interest with correlation coefficients higher than 0.3 but lower than 0.5 [44] were observed, which are noteworthy. OSDI positively correlated with the dendritic cell density (r = 0.429, *p* < 0.001). The NRS positively correlated with corneal staining (r = 0.361, *p* < 0.001) and microneuroma density (r = 0.405, *p* < 0.001) but negatively correlated with the number of nerves (r = −0.337, *p* = 0.001) and nerve density (r = −0.369, *p* < 0.001). WBFPRS showed the same correlations as the NRS, except for the number of nerves, whose correlation did not reach a moderate level. Two positive correlations were shown between the m-SIDEQ and depression (r = 0.307, *p* = 0.003), and dendritic cell density (r = 0.440, *p* < 0.001). In addition, a positive correlation was obtained between corneal staining and dendritic cell density (r = 0.496, *p* < 0.001), while a negative correlation was obtained with corneal tactile sensitivity without anesthesia (r = −0.353, *p* = 0.001). The corneal tactile sensitivity value without anesthesia positively correlated with nerve number (r = 0.328, *p* = 0.001), nerve density (r = 0.313, *p* = 0.002), and nerve branches (r = 0.392, *p* < 0.001).

## 4. Discussion

Chronic ocular pain, as well as DED, negatively affects patients’ quality of life, impacting on their physical, mental, and social functioning [45,46,47]. Diagnosing this type of pain remains a clinical challenge due to the absence of standardized criteria, currently relying mainly on subjective questionnaires [14]. In addition, its underlying pathophysiology is not yet fully elucidated [13]. These factors contribute to the complexity of its clinical management and therapeutic approach. Accordingly, there is a growing interest in the identification and characterization of ocular pain associated with DED that could help its diagnosis and management, as well as establish therapeutic targets for the development of new drugs and more effective therapies. Therefore, in the present study, the LFU of pain-DED patients was clinically characterized by comparing them with no pain-DED patients and healthy controls. Our results showed that the pain-DED group had higher ocular pain symptomatology, along with a reduced tear meniscus, increased corneal and conjunctival staining, and higher Meibomian gland loss. These patients also presented reduced sub-basal corneal nerve density and increased density of dendritic cells and microneuromas.

Participants included in the present study were mostly women, with a 9-year history of DED and moderate ocular pain intensity [48] in the pain-DED group. Our results revealed that ocular pain emerged approximately three years after the onset of other DED-related symptoms, suggesting that these patients initially experienced non-painful ocular symptoms and later progressed and developed ocular pain over time. Moreover, the pain-DED group reported a higher severity and frequency of DED-related symptoms compared with the no pain-DED group. These findings are consistent with previous studies reporting an association between increased DED-related symptoms and ocular pain symptomatology [49,50,51,52]. Additionally, the relationship between DED and ocular pain symptomatology with mental disorders such as depression and anxiety has been previously described by other authors [51,52,53,54,55], which is in line with our results.

Significant alterations were also observed in several ocular surface parameters, including tear meniscus, TBUT, nasal conjunctival staining, corneal staining, and Meibomian gland loss. The pain-DED group presented the smallest tear meniscus and the highest grades of corneal and conjunctival staining and Meibomian gland loss. On the other hand, the no pain-DED group showed the lowest TBUT. These findings suggest that the clinical characteristics of the LFU may differ slightly between DED patients with and without chronic ocular pain. Previous studies have reported differences in the LFU of DED patients [55,56,57,58]; however, the literature specifically addressing LFU characteristics in patients with DED and ocular pain is scarce. Notably, higher corneal staining has been observed in painful DED patients with primary Meibomian gland dysfunction compared with their painless counterparts [49]. In addition, a previous study reported no relationship between ocular pain and TBUT or corneal staining, although their analysis was based on pain severity levels (absence, mild, moderate, and severe) [50], whereas our study compared patients with and without pain, regardless of the pain intensity.

The pain-DED group also presented alterations in the sub-basal corneal nerve plexus, including reduced nerve number, density, and branch points, along with increased microneuroma density. On the other hand, the no pain-DED group showed increased nerve tortuosity compared with controls. Both DED groups showed increased dendritic cell density, particularly the pain-DED group, although the difference between both DED groups did not reach statistical significance. These findings are consistent with previous literature reporting reduced nerve number, density, and branches, as well as increased tortuosity and dendritic cell density in DED [59,60,61,62]. Decreased nerve number, density, branches, and length, as well as increased nerve tortuosity, have also been reported in patients suffering from ocular pain and DED compared with healthy controls [49,62,63], with only statistically significant differences in the microneuroma density observed between painful and painless patients [49,62], which is in line with our results. Similar corneal nerve alterations were identified in a previous study by our group in patients who developed chronic neuropathic ocular pain and DED following laser-assisted in situ keratomileusis [51]. Taken together, these data suggest that reduced nerve number, density, and branch points seem to be related to both inflammatory and pain-related mechanisms in DED, whereas an increased microneuroma density appears to be specifically associated with pain, and dendritic cell density is associated with inflammation. These changes in the corneal nerve plexus morphology could trigger alterations in nerve transmission, which may help explain, at least in part, the presence of ocular pain in some patients with DED.

Additionally, strong and statistically significant correlations were observed among the studied variables. All of them were expected, as they occurred between variables measuring similar aspects: between questionnaires for ocular symptomatology (OSDI and m-SIDEQ; m-SIDEQ and NRS; NRS and WBFPRS), between high- and low-contrast visual acuity, among tear meniscus parameters, and among corneal nerve plexus parameters. Furthermore, numerous moderately significant correlations were also found. For instance, a higher ocular symptomatology was moderately associated with increased corneal staining and higher dendritic cell density. According to previous studies, both corneal staining and dendritic cell density have been related to ocular surface inflammation [64,65], so it can be suggested that ocular symptomatology is influenced by the extent to which the LFU is affected by inflammation. In fact, a positive correlation was observed between corneal staining and dendritic cell density, which can support this relationship. In addition, DED-related symptomatology was found to be associated with emotional disorders such as depression, which is consistent with previous findings [55,66]. Moreover, increased microneuroma density correlated with higher ocular pain intensity, which is consistent with our group comparison results.

Finally, reduced corneal tactile sensitivity, as measured with the Cochet–Bonnet esthesiometer, was associated with increased corneal staining, as well as with reduced nerve number and density, and these, in turn, are correlated with increased DED-related symptoms and ocular pain intensity. In other words, patients reporting higher DED-related symptoms and ocular pain intensity presented increased corneal staining and fewer corneal nerves and required a stronger stimulus with the nylon filament to perceive it. These findings suggest that alterations in the corneal nerve morphology may affect the corneal sensitivity and play a key role in ocular symptomatology in patients with DED.

In summary, the present study provides an overview of the alterations that occur at the clinical level in pain-DED patients. Considering all findings, our results indicate that in some individuals, DED progresses and eventually leads to ocular pain, typically emerging after an average of three years following the onset of DED-related symptoms. Once ocular pain develops and persists over time, these patients tend to experience high severity and frequency of DED-related symptoms, as well as moderate pain symptomatology. In addition, pain-DED patients exhibit ocular surface disruption and alterations in corneal nerve morphology compared to no pain-DED patients and/or controls.

This study has, however, some limitations. First, by using a non-standard diagnostic criteria, including the Schirmer test as a possible sign of the disease and requiring two signs to be present in addition to symptoms, the findings of this study may differ from those that used different diagnostic criteria. Second, this study was restricted to patients suffering from chronic pain, and the specific type of pain (neuropathic, nociceptive, or mixed) of these patients was not considered. Finally, we did not address whether there were differences in the results according to the type of DED (mainly evaporative or aqueous deficient). Exploring the LFU characteristics in patients with different subtypes of DED and/or ocular pain could provide valuable insights into the underlying mechanisms of each condition. Identifying both their shared and distinct features may contribute to the development of more targeted and personalized therapeutic approaches.

## 5. Conclusions

Alterations in the LFU, especially in the morphology of the sub-basal corneal nerves, along with pain clinical questionnaires, can help phenotype patients with chronic ocular pain associated with DED. Specifically, when DED is associated with chronic pain, there is a decreased number and density of corneal nerves, along with an increased density of microneuromas, making in vivo confocal microscopy a useful tool to explain the presence of ocular pain. Therefore, this evaluation might be incorporated into routine clinical practice for patients suffering from ocular pain.

## Figures and Tables

**Table 1:** Demographic characteristics of the three groups.

Demographic Characteristics	Pain-DED	No Pain-DED	Controls	*p*-Value *
Age (years)	60.89 ± 10.06	61.20 ± 13.01	58.87 ± 9.39	0.668
n (%) females/males	24/4 (85.7/14.3)	25/10 (71.4/28.6)	18/12 (60.0/40.0)	0.092
Years with DED-related symptoms	9.34 ± 10.28	9.85 ± 8.94	----	0.637
Years with ocular pain	6.49 ± 6.14	----	----	----

* *p*-values refer to the comparison among the three groups, except for the variable “years with DED-related symptoms”, whose *p*-value corresponds to the comparison between both DED groups. DED: dry eye disease.

## Data Availability

The original contributions presented in this study are included in the article. Further inquiries can be directed to the corresponding author.

## Supplementary Materials

The following supporting information can be downloaded at: https://www.mdpi.com/article/10.3390/jcm14155250/s1, Tables S1–S4 present the comparisons of the symptom questionnaires and clinical tests among the three groups; Tables S5–S9 present all the correlation analyses conducted in this study.

## Abbreviations

The following abbreviations are used in this manuscript:

ANOVA	Analysis of variance
CCLRU	Cornea and Contact Lens Research Unit
CELab	Controlled Environment Laboratory
Controls	Subjects without dry eye disease or ocular pain
DED	Dry eye disease
ETDRS	Early Treatment Diabetic Retinopathy Study
GRC	Global Rating of Change
HADS	Hospital Anxiety and Depression Scale
ICC	Intraclass correlation coefficient
LFU	Lacrimal functional unit
m-SIDEQ	Modified Single-Item score Dry Eye Questionnaire
No pain-DED	Patients with dry eye disease but no pain
NRS	Numerical Rating Scale
OSDI	Ocular Surface Disease Index
Pain-DED	Patients with chronic ocular pain associated with dry eye disease
TBUT	Tear break-up time
WBFPRS	Wong–Baker Faces^®^ Pain-Rating Scale

## References

[1] Stern M.E., Beuerman R.W., Fox R.I., Gao J., Mircheff A.K., Pflugfelder S.C. (1998). The Pathology of Dry Eye: The Interaction between the Ocular Surface and Lacrimal Glands. Cornea.

[2] Craig J.P., Nichols K.K., Akpek E.K., Caffery B., Dua H.S., Joo C.-K., Liu Z., Nelson J.D., Nichols J.J., Tsubota K. (2017). TFOS DEWS II Definition and Classification Report. Ocul. Surf..

[3] Belmonte C., Nichols J.J., Cox S.M., Brock J.A., Begley C.G., Bereiter D.A., Dartt D.A., Galor A., Hamrah P., Ivanusic J.J. (2017). TFOS DEWS II Pain and Sensation Report. Ocul. Surf..

[4] World Health Organization (WHO) (2012). WHO Guidelines on the Pharmacological Treatment of Persisting Pain in Children with Medical Illnesses.

[5] Treede R.D., Rief W., Barke A., Aziz Q., Bennett M.I., Benoliel R., Cohen M., Evers S., Finnerup N.B., First M.B. (2019). Chronic Pain as a Symptom or a Disease: The IASP Classification of Chronic Pain for the International Classification of Diseases (ICD-11). Pain.

[6] Goldberg D.S., McGee S.J. (2011). Pain as a Global Public Health Priority. BMC Public Health.

[7] Tsang A., Von Korff M., Lee S., Alonso J., Karam E., Angermeyer M.C., Borges G.L.G., Bromet E.J., de Girolamo G., de Graaf R. (2008). Common Chronic Pain Conditions in Developed and Developing Countries: Gender and Age Differences and Comorbidity with Depression-Anxiety Disorders. J. Pain.

[8] Henschke N., Kamper S.J., Maher C.G. (2015). The Epidemiology and Economic Consequences of Pain. Mayo Clin. Proc..

[9] Stapleton F., Alves M., Bunya V.Y., Jalbert I., Lekhanont K., Malet F., Na K.S., Schaumberg D., Uchino M., Vehof J. (2017). TFOS DEWS II Epidemiology Report. Ocul. Surf..

[10] Müller L.J., Marfurt C.F., Kruse F., Tervo T.M.T. (2003). Corneal Nerves: Structure, Contents and Function. Exp. Eye Res..

[11] Galor A., Levitt R.C., Felix E.R., Martin E.R., Sarantopoulos C.D. (2015). Neuropathic Ocular Pain: An Important yet Underevaluated Feature of Dry Eye. Eye.

[12] Rusciano D., Bagnoli P., Gallar J., Galor A. (2022). Editorial: Eye Pain: Etiology and Therapeutic Approacheses. Front. Pharmacol..

[13] Nicolle P., Liang H., Reboussin E., Rabut G., Warcoin E., Brignole-Baudouin F., Melik-Parsadaniantz S., Baudouin C., Labbe A., Goazigo A.R. (2018). Le Proinflammatory Markers, Chemokines, and Enkephalin in Patients Suffering from Dry Eye Disease. Int. J. Mol. Sci..

[14] Jacobs D.S. (2017). Diagnosis and Treatment of Ocular Pain: The Ophthalmologist’s Perspective. Curr. Ophthalmol. Rep..

[15] Blanco-Vázquez M., Vázquez A., Fernández I., Novo-Diez A., Martínez-Plaza E., García-Vázquez C., González-García M.J., Sobas E.M., Calonge M., Enríquez-de-Salamanca A. (2022). Inflammation-Related Molecules in Tears of Patients with Chronic Ocular Pain and Dry Eye Disease. Exp. Eye Res..

[16] Wong-Baker FACES Foundation Wong-Baker FACES® Pain Rating Scale. https://wongbakerfaces.org/.

[17] Galor A., Hamrah P., Haque S., Attal N., Labetoulle M. (2022). Understanding Chronic Ocular Surface Pain: An Unmet Need for Targeted Drug Therapy. Ocul. Surf..

[18] Calonge M., Pinto-Fraga J., González-García M.J., Enríquez-de-Salamanca A., López-de la Rosa A., Fernández I., López-Miguel A. (2017). Effects of the External Environment on Dry Eye Disease. Int. Ophthalmol. Clin..

[19] López-Miguel A., Tesón M., Martín-Montañez V., Enríquez-de-Salamanca A., Stern M.E., Calonge M., González-García M.J. (2014). Dry Eye Exacerbation in Patients Exposed to Desiccating Stress under Controlled Environmental Conditions. Am. J. Ophthalmol..

[20] Miller K.L., Walt J.G., Mink D.R., Satram-Hoang S., Wilson S.E., Perry H.D., Asbell P.A., Pflugfelder S.C. (2010). Minimal Clinically Important Difference for the Ocular Surface Disease Index. Arch. Ophthalmol..

[21] Simmons P.A., Vehige J.G., Carlisle C., Felix C. Comparison of Dry Eye Signs in Self-Described Mild and Moderate Patients. Proceedings of the ARVO Annual Meeting.

[22] Ferreira-Valente M.A., Pais-Ribeiro J.L., Jensen M.P. (2011). Validity of Four Pain Intensity Rating Scales. Pain.

[23] Zigmond A.S., Snaith R.P. (1983). The Hospital Anxiety and Depression Scale. Acta Psychiatr. Scand..

[24] Zhou S., Li Y., Lu A.T.H., Liu P., Tang M., Yiu S.C., Huang D. (2009). Reproducibility of Tear Meniscus Measurement by Fourier-Domain Optical Coherence Tomography: A Pilot Study. Ophthalmic Surg. Lasers Imaging.

[25] Tukenmez-Dikmen N., Yildiz E.H., Imamoglu S., Turan-Vural E., Sevim M.S. (2016). Correlation of Dry Eye Workshop Dry Eye Severity Grading System with Tear Meniscus Measurement by Optical Coherence Tomography and Tear Osmolarity. Eye Contact Lens.

[26] Yokoi N., Takehisa Y., Kinoshita S. (1996). Correlation of Tear Lipid Layer Interference Patterns with the Diagnosis and Severity of Dry Eye. Am. J. Ophthalmol..

[27] Efron N. (1998). Grading Scales for Contact Lens Complications. Ophthalmic Physiol. Opt..

[28] Terry R.L., Schnider C.M., Holden B.A., Cornish R., Grant T., Sweeney D., La Hood D., Back A. (1993). CCLRU Standards for Success of Daily and Extended Wear Contact Lenses. Optom. Vis. Sci..

[29] Bron A.J., Evans V.E., Smith J.A. (2003). Grading of Corneal and Conjunctival Staining in the Context of Other Dry Eye Tests. Cornea.

[30] Korb D.R., Herman J.P., Greiner J.V., Scaffidi R.C., Finnemore V.M., Exford J.M., Blackie C.A., Douglass T. (2005). Lid Wiper Epitheliopathy and Dry Eye Symptoms. Eye Contact Lens.

[31] Bron A.J., Benjamin L., Snibson G.R. (1991). Meibomian Gland Disease. Classification and Grading of Lid Changes. Eye.

[32] Shimazaki J., Goto E., Ono M., Shimmura S., Tsubota K. (1998). Meibomian Gland Dysfunction in Patients with Sjögren Syndrome. Ophthalmology.

[33] Pult H., Riede-Pult B. (2013). Comparison of Subjective Grading and Objective Assessment in Meibography. Cont. Lens Anterior Eye.

[34] Belmonte C., Acosta M.C., Schmelz M., Gallar J. (1999). Measurement of Corneal Sensitivity to Mechanical and Chemical Stimulation with a CO2 Esthesiometer. Investig. Ophthalmol. Vis. Sci..

[35] Yarnitsky D., Ochoa J.L. (1990). Studies of Heat Pain Sensation in Man: Perception Thresholds, Rate of Stimulus Rise and Reaction Time. Pain.

[36] Acosta M.C., Belmonte C., Gallar J. (2001). Sensory Experiences in Humans and Single-Unit Activity in Cats Evoked by Polymodal Stimulation of the Cornea. J. Physiol..

[37] Acosta M.C., Berenguer-Ruiz L., García-Gálvez A., Perea-Tortosa D., Gallar J., Belmonte C. (2005). Changes in Mechanical, Chemical, and Thermal Sensitivity of the Cornea after Topical Application of Nonsteroidal Anti-Inflammatory Drugs. Investig. Ophthalmol. Vis. Sci..

[38] Chao C., Stapleton F., Badarudin E., Golebiowski B. (2015). Ocular Surface Sensitivity Repeatability with Cochet-Bonnet Esthesiometer. Optom. Vis. Sci..

[39] Kamper S.J., Maher C.G., Mackay G. (2009). Global Rating of Change Scales: A Review of Strengths and Weaknesses and Considerations for Design. J. Man. Manip. Ther..

[40] Oliveira-Soto L., Efron N. (2001). Morphology of Corneal Nerves Using Confocal Microscopy. Cornea.

[41] Faul F., Erdfelder E., Lang A.G., Buchner A. (2007). G*Power 3: A Flexible Statistical Power Analysis Program for the Social, Behavioral, and Biomedical Sciences. Behav. Res. Methods.

[42] Cohen J. (1988). Statistical Power Analysis for the Behavioral Sciences.

[43] IBM Corp IBM SPSS Statistics. https://www.ibm.com/es-es/products/spss-statistics.

[44] Ratner B. (2009). The Correlation Coefficient: Its Values Range between 1/1, or Do They. J. Targeting Meas. Anal. Mark..

[45] Patel S., Felix E.R., Levitt R.C., Sarantopoulos C.D., Galor A. (2019). Dysfunctional Coping Mechanisms Contribute to Dry Eye Symptoms. J. Clin. Med..

[46] Fine P.G. (2011). Long-Term Consequences of Chronic Pain: Mounting Evidence for Pain as a Neurological Disease and Parallels with Other Chronic Disease States. Pain Med..

[47] Miljanović B., Dana R., Sullivan D.A., Schaumberg D.A. (2007). Impact of Dry Eye Syndrome on Vision-Related Quality of Life. Am. J. Ophthalmol..

[48] Breivik H., Borchgrevink P.C., Allen S.M., Rosseland L.A., Romundstad L., Breivik Hals E.K., Kvarstein G., Stubhaug A. (2008). Assessment of Pain. Br. J. Anaesth..

[49] Guerrero-Moreno A., Liang H., Moreau N., Luzu J., Rabut G., Parsadaniantz S.M., Labbé A., Baudouin C., Goazigo A.R. (2021). Le Corneal Nerve Abnormalities in Painful Dry Eye Disease Patients. Biomedicines.

[50] Satitpitakul V., Kheirkhah A., Crnej A., Hamrah P., Dana R. (2017). Determinants of Ocular Pain Severity in Patients with Dry Eye Disease. Am. J. Ophthalmol..

[51] Vázquez A., Blanco-Vázquez M., Martínez-Plaza E., Sobas E.M., González-García M.J., López-Miguel A., Ortega E., Enríquez-de-Salamanca A., Calonge M. (2025). Corneal Sensory Changes and Nerve Plexus Abnormalities in Chronic Neuropathic Ocular Pain and Dry Eye Post-Refractive Surgery. Am. J. Ophthalmol..

[52] Siedlecki A.N., Smith S.D., Siedlecki A.R., Hayek S.M., Sayegh R.R. (2020). Ocular Pain Response to Treatment in Dry Eye Patients. Ocul. Surf..

[53] Vázquez A., Martínez-Plaza E., Fernández I., Sobas E.M., González-García M.J., Enríquez-de-Salamanca A., Ortega E., López-Miguel A., Calonge M. (2022). Phenotypic Characterization of Patients Developing Chronic Dry Eye and Pain after Refractive Surgery: A Cross-Sectional Study. Ocul. Surf..

[54] Galor A., Felix E.R., Feuer W., Shalabi N., Martin E.R., Margolis T.P., Sarantopoulos C.D., Levitt R.C. (2015). Dry Eye Symptoms Align More Closely to Non-Ocular Conditions than to Tear Film Parameters. Br. J. Ophthalmol..

[55] Wu M., Liu X., Han J., Shao T., Wang Y. (2019). Association Between Sleep Quality, Mood Status, and Ocular Surface Characteristics in Patients with Dry Eye Disease. Cornea.

[56] Singh A., Vanathi M., Kishore A., Gupta N., Tandon R. (2019). Evaluation of Strip Meniscometry, Tear Meniscus Height and Depth in the Diagnosis of Dry Eye Disease in Asian Indian Eyes. Ocul. Surf..

[57] Ye F., Jiang F., Lu Y., Xue C., Zhu X., Wu Y., Huang Z. (2019). Objective Optical Assessment of Tear-Film Quality Dynamics in Patients with Meibomian Gland Dysfunction and Aqueous-Deficient Dry Eye Optical Quality Changes in Different Dry Eye Subtypes. Indian J. Ophthalmol..

[58] Ayaki M., Kawashima M., Uchino M., Tsubota K., Negishi K. (2018). Gender Differences in Adolescent Dry Eye Disease: A Health Problem in Girls. Int. J. Ophthalmol..

[59] Labbé A., Alalwani H., Van Went C., Brasnu E., Georgescu D., Baudouin C. (2012). The Relationship between Subbasal Nerve Morphology and Corneal Sensation in Ocular Surface Disease. Investig. Ophthalmol. Vis. Sci..

[60] Ma B., Xie J., Yang T., Su P., Liu R., Sun T., Zhou Y., Wang H., Feng X., Ma S. (2021). Quantification of Increased Corneal Subbasal Nerve Tortuosity in Dry Eye Disease and Its Correlation with Clinical Parameters. Transl. Vis. Sci. Technol..

[61] Villani E., Ceresara G., Beretta S., Magnani F., Viola F., Ratiglia R. (2011). In Vivo Confocal Microscopy of Meibomian Glands in Contact Lens Wearers. Investig. Ophthalmol. Vis. Sci..

[62] Moein H.R., Akhlaq A., Dieckmann G., Abbouda A., Pondelis N., Salem Z., Müller R.T., Cruzat A., Cavalcanti B.M., Jamali A. (2020). Visualization of Microneuromas by Using in Vivo Confocal Microscopy: An Objective Biomarker for the Diagnosis of Neuropathic Corneal Pain?. Ocul. Surf..

[63] Zhang Y., Wu Y., Li W., Huang X. (2022). Semiautomated and Automated Quantitative Analysis of Corneal Sub-Basal Nerves in Patients with DED with Ocular Pain Using IVCM. Front. Med..

[64] Shetty R., Deshpande K., Deshmukh R., Jayadev C., Shroff R. (2016). Bowman Break and Subbasal Nerve Plexus Changes in a Patient with Dry Eye Presenting with Chronic Ocular Pain and Vitamin D Deficiency. Cornea.

[65] Hamrah P., Liu Y., Zhang Q., Dana M.R. (2003). Alterations in Corneal Stromal Dendritic Cell Phenotype and Distribution in Inflammation. Arch. Ophthalmol..

[66] He Q., Chen Z., Xie C., Liu L., Yang H., Wei R. (2022). Relationship Between Dry Eye Disease and Emotional Disorder: The Mediating Effect of Health Anxiety. Front. Public Health.

