# Supplementary material for: Clinical Characterization of the Lacrimal Functional Unit in Patients with Chronic Ocular Pain Associated with Dry Eye Disease
Source: J Clin Med. 2025 Jul 24;14(15):5250. doi: 10.3390/jcm14155250 (PMC12347152; doi:10.3390/jcm14155250)
Supplement: Supplementary file 1 [file jcm-14-05250-s001.zip › jcm-3698768-supplementary.pdf]

Supplementary S1

The following tables present the comparisons of the symptom questionnaires and clinical tests among the three groups.

Table S1. Comparison of the symptom questionnaire scores among the three groups.

Questionnaires	Pain-DED	No pain-DED	Controls	P-value*
OSDI (0-100 score)	44.72 ± 19.82	28.99 ± 14.60[a]	3.21 ± 2.97[a,b]	**<0.001**
m-SIDEQ (0-28 score)	16.21 ± 3.47	9.66 ± 3.59[a]	3.87 ± 2.90[a,b]	**<0.001**
NRS (0-10 score)	6.54 ± 1.88	0.23 ± 0.41[a]	0.03 ± 0.18[a,b]	**<0.001**
WBFPRS (0-10 score)	6.36 ± 2.06	0.17 ± 0.57[a]	0.13 ± 0.51[a]	**<0.001**
Anxiety subscale – HADS (0-21 score)	6.32 ± 3.91	5.91 ± 3.29	4.47 ± 2.53	0.094
Depression subscale – HADS (0-21 score)	3.89 ± 3.17	3.20 ± 3.20	1.70 ± 1.53[a]	**0.026**

*P-values refer to the comparison among the three groups. P-values ≤ 0.05 were considered statistically significant and are shown in bold. [a] Significant post hoc difference vs. pain-DED. [b] Significant post hoc difference vs. no pain-DED. DED: dry eye disease; HADS: Hospital Anxiety and Depression Scale; m-SIDEQ: modified Single-Item score Dry Eye Questionnaire; NRS: Numerical Rating Scale; OSDI: Ocular Surface Disease Index; WBFPRS: Wong-Baker Faces® Pain Rating Scale.

Table S2. Comparison of the visual function assessment among the three groups.

Variables	Pain-DED	No pain-DED	Controls	P-value*
High contrast visual acuity (LogMAR)	0.04 ± 0.24	0.05 ± 0.16	0.04 ± 0.17	0.703
Low contrast visual acuity (LogMAR)	0.31 ± 0.20	0.39 ± 0.18	0.34 ± 0.17	0.195
Total HOA RMS (μm)	0.14 ± 0.10	0.13 ± 0.07	0.12 ± 0.06	0.716

*P-values refer to the comparison among the three groups. DED: dry eye disease; total HOA RMS: root mean square of the total higher-order aberrations (from third to sixth order).

Table S3. Comparison of the ocular surface parameters among the three groups.

Variables	Pain-DED	No pain-DED	Controls	P-value*
Tear meniscus height (μm)	159.87 ± 95.35	246.03 ± 176.20	201.16 ± 63.14[a]	**0.043**
Tear meniscus depth (μm)	128.33 ± 54.77	186.02 ± 120.79	174.04 ± 54.27[a]	**0.018**
Tear meniscus area (μm^2)	12187.18 ± 14518.64	27096.84 ± 35063.04	17601.03 ± 10548.29[a]	**0.034**
Tear meniscus angle (grades)	45.04 ± 8.16	41.37 ± 10.36	42.48 ± 8.24	0.347
Lipid layer pattern (1-5 scale)	3.00 [2.00, 3.00]	2.00 [2.00, 3.00]	2.00 [2.00, 3.00]	0.453
Bulbar conjunctival hyperemia (0-4 scale)	1.50 [1.00, 2.00]	1.00 [1.00, 2.00]	1.00 [1.00, 2.00]	0.757
Tarsal conjunctival hyperemia (0-4 scale)	2.00 [1.00, 3.00]	2.00 [1.00, 3.00]	2.00 [1.00, 2.00]	0.599
Nasal lid parallel conjunctival folds	1.64 ± 1.03	1.63 ± 0.84	1.87 ± 0.68	0.377
Temporal lid parallel conjunctival folds	1.82 ± 0.91	1.49 ± 1.01	1.63 ± 0.85	0.484
TBUT (seconds)	3.87 ± 1.44	2.95 ± 0.94[a]	4.86 ± 2.12[b]	**<0.001**
Corneal staining (0-20 CCLRU scale)	4.14 ± 2.55	3.43 ± 2.91	1.23 ± 1.10[a,b]	**<0.001**
Corneal staining (0-5 Oxford scale)	1.00 [1.00, 2.00]	1.00 [1.00, 2.00]	0.00 [0.00, 0.00][a,b]	**<0.001**
Nasal conjunctival staining (0-5 scale)	2.00 [1.00, 2.00]	1.00 [1.00, 2.00]	1.00 [0.00, 2.00][a]	**0.032**
Temporal conjunctival staining (0-5 scale)	1.00 [0.00, 1.00]	1.00 [0.00, 1.00]	0.00 [0.00, 1.00]	0.117
Total conjunctival staining (0-5 scale)	1.50 [0.50, 1.50]	1.00 [0.50, 1.50]	1.00 [0.37, 1.50]	0.051
Lid wiper epitheliopathy (0-3 scale)	0.50 [0.50, 1.00]	0.50 [0.00, 1.00]	0.50 [0.00, 1.00]	0.745
Lid margin (0-4 scale)	2.00 [1.00, 2.00]	2.00 [1.00, 2.00]	2.00 [1.00, 2.00]	0.489

Quality of meibum secretion (0-3 scale)	1.00 [1.00, 2.00]	2.00 [1.00, 2.00]	1.00 [0.00, 2.00]	0.064
Expressibility of meibum secretion (0-3 scale)	1.50 [1.00, 2.00]	1.00 [1.00, 2.00]	1.00 [0.00, 2.00]	**0.036**
Number of obstructed Meibomian glands	0.29 ± 0.60	0.14 ± 0.43	0.07 ± 0.37	0.114
Lower eyelid MGL (0-4 scale)	2.00 [1.00, 3.00]	1.00 [1.00, 2.00]	1.00 [0.00, 1.00][a,b]	**0.003**
Upper eyelid MGL (0-4 scale)	2.00 [2.00, 3.00]	2.00 [1.00, 3.00]	2.00 [1.00, 2.00][a]	**0.025**
Schirmer with anesthesia (mm)	9.68 ± 7.12	9.49 ± 9.01	9.03 ± 5.37	0.752

*P-values refer to the comparison among the three groups. P-values ≤ 0.05 were considered statistically significant and are shown in bold. [a] Significant post hoc difference vs. pain-DED. [b] Significant post hoc difference vs. no pain-DED. CCLRU: Cornea and Contact Lens Research Unit; DED: dry eye disease; TBUT: tear break-up time; MGL: Meibomian gland loss.

Table S4. Comparison of the corneal nerve plexus parameters among the three groups.

Variables	Pain-DED	No pain-DED	Controls	P-value*
Corneal sensitivity - Belmonte non-contact esthesiometer				
Mechanical sensitivity threshold (mL/min)	98.86 ± 45.58	103.23 ± 46.09	126.33 ± 51.49	0.099
Thermal-cold sensitivity threshold (ºC)	-2.37 ± 1.08	-2.02 ± 1.16	-1.83 ± 1.02	0.233
Thermal-hot sensitivity threshold (ºC)	2.00 ± 1.23	1.55 ± 1.22	1.38 ± 1.09	0.104
Corneal tactile sensitivity - Cochet-Bonnet esthesiometer				
Tactile sensitivity without anesthesia (mm)	52.50 ± 13.78	55.14 ± 6.91	58.00 ± 3.62	0.235
Tactile sensitivity with anesthesia (mm)	12.32 ± 21.96	13.43 ± 20.82	17.50 ± 24.27	0.953
Anesthetic challenge test				
GRC (-5 - +5 scale)	1.82 ± 2.20	1.07 ± 1.85	0.63 ± 2.25	0.097
In vivo confocal microscopy				
Number of nerves (n/mm^2)	43.23 ± 14.03	52.92 ± 20.38	62.21 ± 20.93[a]	**0.001**
Nerve density (µm/mm^2)	9807.59 ± 3737.81	12146.41 ± 4212.82	14029.55 ± 3617.80[a]	**<0.001**
Nerve length (µm/mm^2)	1411.81 ± 281.01	1461.22 ± 248.22	1464.16 ± 165.78	0.675
Nerve tortuosity (0-4 scale)	2.76 ± 0.68	2.93 ± 0.61	2.59 ± 0.57[b]	**0.040**
Image reflectivity (Gray units)	99.47 ± 8.55	103.57 ± 12.19	100.73 ± 13.17	0.463
Density of nerve branch points (n/mm^2)	21.09 ± 14.51	30.71 ± 23.59	39.48 ± 27.65[a]	**0.022**
Dendritic cell density (n/mm^2)	93.45 ± 110.54	60.89 ± 52.39	13.22 ± 15.71[a,b]	**<0.001**
Microneuroma density (n/mm^2)	2.01 ± 3.57	0.33 ± 1.03[a]	0.14 ± 0.37[a]	**<0.001**

*P-values refer to the comparison among the three groups. P-values ≤ 0.05 were considered statistically significant and are shown in bold. [a] Significant post hoc difference vs. pain-DED. [b] Significant post hoc difference vs. no pain-DED. DED: dry eye disease; GRC: Global Rating of Change.

Supplementary S2

The following tables present all the correlation analyses conducted in this study.

Table S5. Correlations with symptomatology.

Variables		OSDI	NRS	WBFPRS	m-SIDEQ	Anxiety subscale (HADS)	Depression subscale (HADS)
OSDI	r	1.000	**0.577**	**0.529**	**0.822**	0.182	**0.270**
	p-value		**0.000**	**0.000**	**0.000**	0.081	**0.009**
NRS	r	**0.577**	1.000	**0.928**	**0.706**	0.118	**0.243**
	p-value	**0.000**		**0.000**	**0.000**	0.261	**0.019**
WBFPRS	r	**0.529**	**0.928**	1.000	**0.669**	0.129	0.197
	p-value	**0.000**	**0.000**		**0.000**	0.218	0.058
m-SIDEQ	r	**0.822**	**0.706**	**0.669**	1.000	**0.247**	**0.307**
	p-value	**0.000**	**0.000**	**0.000**		**0.017**	**0.003**
Anxiety	r	0.182	0.118	0.129	**0.247**	1.000	**0.672**
	p-value	0.081	0.261	0.218	**0.017**		**0.000**
Depression	r	**0.270**	**0.243**	0.197	**0.307**	**0.672**	1.000
	p-value	**0.009**	**0.019**	0.058	**0.003**	**0.000**	
High contrast VA	r	0.059	-0.033	-0.041	0.067	0.124	0.097
	p-value	0.580	0.758	0.701	0.529	0.240	0.360
Low contrast VA	r	0.025	-0.089	-0.114	-0.058	0.031	0.072
	p-value	0.815	0.407	0.290	0.590	0.773	0.503
Total HOA RMS	r	0.158	0.089	0.092	0.170	-0.033	0.049
	p-value	0.134	0.401	0.386	0.108	0.755	0.647
Meniscus area	r	-0.204	**-0.254**	**-0.285**	**-0.225**	**-0.221**	-0.154
	p-value	0.065	**0.022**	**0.009**	**0.042**	**0.046**	0.168
Meniscus depth	r	**-0.231**	**-0.243**	**-0.273**	**-0.235**	-0.211	-0.099
	p-value	**0.037**	**0.028**	**0.013**	**0.034**	0.057	0.374
Meniscus height	r	-0.170	**-0.261**	**-0.288**	-0.187	**-0.228**	-0.169
	p-value	0.127	**0.018**	**0.009**	0.093	**0.040**	0.128
Meniscus angle	r	-0.002	0.133	0.115	0.009	0.186	0.189
	p-value	0.982	0.233	0.302	0.937	0.093	0.090
Nasal LIPCOF	r	-0.038	-0.055	-0.027	-0.128	-0.023	0.008
	p-value	0.716	0.598	0.794	0.222	0.823	0.941
Temporal LIPCOF	r	0.070	0.107	0.155	0.009	0.180	0.117
	p-value	0.505	0.307	0.138	0.930	0.085	0.263
TBUT	r	**-0.205**	0.047	0.088	-0.009	-0.049	0.034
	p-value	**0.049**	0.652	0.399	0.929	0.639	0.744
Corneal staining	r	**0.514**	**0.361**	**0.317**	**0.504**	0.186	**0.272**
	p-value	**0.000**	**0.000**	**0.002**	**0.000**	0.075	**0.008**
Obstructed MG	r	0.148	**0.229**	0.180	0.187	0.063	0.036
	p-value	0.156	**0.027**	0.083	0.072	0.550	0.731
Schirmer	r	-0.008	-0.013	-0.033	0.058	0.020	0.083
	p-value	0.942	0.900	0.756	0.581	0.852	0.430
Mechanical sensitivity	r	**-0.216**	-0.184	-0.092	**-0.265**	0.099	0.038
	p-value	**0.039**	0.079	0.384	**0.011**	0.350	0.720
Cold sensitivity	r	**-0.242**	-0.114	-0.154	**-0.304**	-0.023	-0.130
	p-value	**0.021**	0.281	0.146	**0.003**	0.831	0.221
Hot sensitivity	r	**0.255**	0.154	0.186	**0.307**	0.113	0.189
	p-value	**0.015**	0.145	0.078	**0.003**	0.285	0.073

Tactile sensitivity without anesthesia	r	-0.198	-0.115	-0.130	-0.180	-0.139	-0.132
	p-value	0.057	0.271	0.215	0.084	0.185	0.206
Anesthetic challenge test	r	**0.229**	0.160	**0.189**	**0.256**	0.085	0.068
	p-value	**0.027**	0.125	**0.070**	**0.013**	0.418	0.515
Tactile sensitivity with anesthesia	r	0.092	0.045	-0.006	0.085	0.029	0.070
	p-value	0.378	0.670	0.956	0.419	0.782	0.503
Nerve number	r	**-0.257**	**-0.337**	**-0.291**	**-0.301**	-0.004	-0.019
	p-value	**0.013**	**0.001**	**0.005**	**0.004**	0.971	0.854
Nerve density	r	**-0.249**	**-0.369**	**-0.335**	**-0.275**	0.091	0.048
	p-value	**0.017**	**0.000**	**0.001**	**0.008**	0.387	0.649
Nerve length	r	-0.009	-0.061	-0.099	0.035	0.113	0.085
	p-value	0.934	0.564	0.345	0.737	0.283	0.418
Nerve reflectivity	r	0.034	-0.055	-0.125	-0.040	0.138	0.091
	p-value	0.745	0.606	0.237	0.708	0.191	0.387
Microneuromas	r	**0.219**	**0.405**	**0.416**	**0.293**	**0.253**	0.150
	p-value	**0.036**	**0.000**	**0.000**	**0.005**	**0.015**	0.154
Dendritic cells	r	**0.429**	0.132	0.130	**0.440**	0.099	0.115
	p-value	**0.000**	0.210	0.215	**0.000**	0.347	0.276
Nerve branches	r	-0.187	**-0.261**	**-0.240**	**-0.252**	-0.037	0.011
	p-value	0.074	**0.012**	**0.021**	**0.015**	0.725	0.915
Nerve tortuosity	r	0.153	0.083	0.048	0.107	0.182	0.164
	p-value	0.146	0.430	0.648	0.310	0.083	0.119

P-values ≤ 0.05 were considered statistically significant and are shown in bold. HADS: Hospital Anxiety and Depression Scale; LIPCOF: lid parallel conjunctival folds; MG: Meibomian glands; m-SIDEQ: modified Single-Item score Dry Eye Questionnaire; NRS: Numerical Rating Scale; OSDI: Ocular Surface Disease Index; r: correlation coefficient; TBUT: tear break-up time; total HOA RMS: root mean square of the total higher-order aberrations (from third to sixth order); VA: visual acuity; WBFPRS: Wong-Baker FACES® Pain Rating Scale.

Table S6. Correlations with visual function and tear meniscus parameters.

**Variables**		**High contrast VA**	**Low contrast VA**	**Total HOA RMS**	**Meniscus area**	**Meniscus depth**	**Meniscus height**	**Meniscus angle**
OSDI	r	0.059	0.025	0.158	-0.204	**-0.231**	-0.170	-0.002
	p-value	0.580	0.815	0.134	0.065	**0.037**	0.127	0.982
NRS	r	-0.033	-0.089	0.089	**-0.254**	**-0.243**	**-0.261**	0.133
	p-value	0.758	0.407	0.401	**0.022**	**0.028**	**0.018**	0.233
WBFPRS	r	-0.041	-0.114	0.092	**-0.285**	**-0.273**	**-0.288**	0.115
	p-value	0.701	0.290	0.386	**0.009**	**0.013**	**0.009**	0.302
m-SIDEQ	r	0.067	-0.058	0.170	**-0.225**	**-0.235**	-0.187	0.009
	p-value	0.529	0.590	0.108	**0.042**	**0.034**	0.093	0.937
Anxiety	r	0.124	0.031	-0.033	**-0.221**	-0.211	**-0.228**	0.186
	p-value	0.240	0.773	0.755	**0.046**	0.057	**0.040**	0.093
Depression	r	0.097	0.072	0.049	-0.154	-0.099	-0.169	0.189
	p-value	0.360	0.503	0.647	0.168	0.374	0.128	0.090
High contrast VA	r	1.000	**0.785**	**0.242**	0.100	0.134	0.107	0.027
	p-value		**0.000**	**0.023**	0.375	0.232	0.343	0.814
Low contrast VA	r	**0.785**	1.000	0.137	**0.227**	0.221	**0.226**	0.028
	p-value	**0.000**		0.210	**0.046**	0.051	**0.046**	0.807
Total HOA RMS	r	**0.242**	0.137	1.000	-0.068	-0.055	-0.060	0.040
	p-value	**0.023**	0.210		0.548	0.623	0.594	0.723
Meniscus area	r	0.100	**0.227**	-0.068	1.000	**0.954**	**0.961**	**-0.477**
	p-value	0.375	**0.046**	0.548		**0.000**	**0.000**	**0.000**
Meniscus depth	r	0.134	0.221	-0.055	**0.954**	1.000	**0.877**	**-0.402**
	p-value	0.232	0.051	0.623	**0.000**		**0.000**	**0.000**
Meniscus height	r	0.107	**0.226**	-0.060	**0.961**	**0.877**	1.000	**-0.581**
	p-value	0.343	**0.046**	0.594	**0.000**	**0.000**		**0.000**
Meniscus angle	r	0.027	0.028	0.040	**-0.477**	**-0.402**	**-0.581**	1.000
	p-value	0.814	0.807	0.723	**0.000**	**0.000**	**0.000**	
Nasal LIPCOF	r	-0.052	0.044	-0.115	**0.372**	**0.388**	**0.337**	**-0.271**
	p-value	0.624	0.683	0.279	**0.001**	**0.000**	**0.002**	**0.014**
Temporal LIPCOF	r	0.136	0.114	0.069	0.214	**0.241**	0.195	-0.182
	p-value	0.198	0.292	0.516	0.054	**0.029**	0.078	0.102
TBUT	r	0.159	-0.019	-0.072	0.064	0.119	0.048	0.002
	p-value	0.132	0.860	0.495	0.568	0.288	0.670	0.987
Corneal staining	r	0.032	-0.044	**0.207**	**-0.374**	**-0.375**	**-0.365**	0.182
	p-value	0.765	0.681	**0.049**	**0.001**	**0.001**	**0.001**	0.102
Obstructed MG	r	0.170	0.122	0.191	-0.045	-0.014	-0.060	0.168
	p-value	0.107	0.256	0.070	0.685	0.904	0.593	0.131
Schirmer	r	-0.014	0.074	**0.213**	**0.313**	**0.275**	**0.295**	-0.140
	p-value	0.892	0.491	**0.042**	**0.004**	**0.012**	**0.007**	0.208
Mechanical sensitivity	r	**0.247**	0.122	0.060	0.079	0.139	0.083	-0.076
	p-value	**0.019**	0.262	0.577	0.482	0.216	0.461	0.501

Cold sensitivity	r	-0.159	-0.143	-0.104	0.042	0.017	0.030	0.122
	p-value	0.136	0.190	0.331	0.706	0.880	0.793	0.277
Hot sensitivity	r	0.014	0.038	0.097	-0.093	-0.070	-0.101	-0.053
	p-value	0.894	0.729	0.365	0.410	0.533	0.369	0.635
Tactile sensitivity without anesthesia	r	-0.094	-0.082	-0.099	0.184	0.158	0.175	-0.122
	p-value	0.377	0.448	0.352	0.098	0.156	0.117	0.273
Anesthetic challenge test	r	0.100	0.015	-0.022	-0.073	-0.071	-0.044	-0.087
	p-value	0.344	0.888	0.838	0.515	0.524	0.697	0.439
Tactile sensitivity with anesthesia	r	**0.302**	**0.287**	**0.278**	0.165	0.129	0.168	-0.136
	p-value	**0.004**	**0.007**	**0.008**	0.139	0.247	0.131	0.223
Nerve number	r	-0.076	-0.040	-0.073	**0.254**	**0.274**	**0.228**	-0.140
	p-value	0.476	0.715	0.494	**0.022**	**0.013**	**0.041**	0.213
Nerve density	r	-0.031	-0.048	-0.169	**0.241**	**0.264**	**0.225**	-0.104
	p-value	0.773	0.658	0.112	**0.030**	**0.017**	**0.044**	0.353
Nerve length	r	0.096	-0.014	-0.034	0.075	0.070	0.086	-0.030
	p-value	0.367	0.894	0.750	0.506	0.533	0.446	0.790
Image reflectivity	r	0.181	0.172	0.135	-0.206	-0.159	-0.186	-0.036
	p-value	0.088	0.112	0.203	0.065	0.156	0.096	0.748
Microneuromas	r	0.100	0.101	-0.012	-0.099	-0.120	-0.069	0.013
	p-value	0.348	0.354	0.909	0.381	0.288	0.541	0.910
Dendritic cells	r	-0.121	-0.109	0.037	-0.190	-0.216	-0.141	0.074
	p-value	0.257	0.314	0.732	0.089	0.053	0.210	0.514
Nerve branches	r	-0.084	-0.047	-0.062	**0.274**	**0.296**	**0.242**	-0.112
	p-value	0.434	0.666	0.561	**0.013**	**0.007**	**0.030**	0.320
Nerve tortuosity	r	0.098	0.124	0.056	0.196	0.189	0.207	-0.194
	p-value	0.358	0.251	0.602	0.079	0.091	0.063	0.082

P-values ≤ 0.05 were considered statistically significant and are shown in bold. LIPCOF: lid parallel conjunctival folds; MG: Meibomian glands; m-SIDEQ: modified Single-Item score Dry Eye Questionnaire; NRS: Numerical Rating Scale; OSDI: Ocular Surface Disease Index; r: correlation coefficient; TBUT: tear break-up time; total HOA RMS: root mean square of the total higher-order aberrations (from third to sixth order); VA: visual acuity; WBFPRS: Wong-Baker FACES® Pain Rating Scale.

Table S7. Correlations with slip-lamp parameters and Schirmer test.

Variables		Nasal LIPCOF	Temporal LIPCOF	TBUT	Corneal staining	Obstructed MG	Schirmer
OSDI	r	-0.038	0.070	**-0.205**	**0.514**	0.148	-0.008
	p-value	0.716	0.505	**0.049**	**0.000**	0.156	0.942
NRS	r	-0.055	0.107	0.047	**0.361**	**0.229**	**0.229**
	p-value	0.598	0.307	0.652	**0.000**	**0.027**	**0.027**
WBFPRS	r	-0.027	0.155	0.088	**0.317**	0.180	-0.033
	p-value	0.794	0.138	0.399	**0.002**	0.083	0.756
m-SIDEQ	r	-0.128	0.009	-0.009	**0.504**	0.187	0.058
	p-value	0.222	0.930	0.929	**0.000**	0.072	0.581
Anxiety	r	-0.023	0.180	-0.049	0.186	0.063	0.020
	p-value	0.823	0.085	0.639	0.075	0.550	0.852
Depression	r	0.008	0.117	0.034	**0.272**	0.036	0.083
	p-value	0.941	0.263	0.744	**0.008**	0.731	0.430
High contrast VA	r	-0.052	0.136	0.159	0.032	0.170	-0.014
	p-value	0.624	0.198	0.132	0.765	0.107	0.892
Low contrast VA	r	0.044	0.114	-0.019	-0.044	0.122	0.074
	p-value	0.683	0.292	0.860	0.681	0.256	0.491
Total HOA RMS	r	-0.115	0.069	-0.072	**0.207**	0.191	**0.213**
	p-value	0.279	0.516	0.495	**0.049**	0.070	**0.042**
Meniscus area	r	**0.372**	0.214	0.064	**-0.374**	-0.045	**0.313**
	p-value	**0.001**	0.054	0.568	**0.001**	0.685	**0.004**
Meniscus depth	r	**0.388**	**0.241**	0.119	**-0.375**	-0.014	**0.275**
	p-value	**0.000**	**0.029**	0.288	**0.001**	0.904	**0.012**
Meniscus height	r	**0.337**	0.195	0.048	**-0.365**	-0.060	**0.295**
	p-value	**0.002**	0.078	0.670	**0.001**	0.593	**0.007**
Meniscus angle	r	**-0.271**	-0.182	0.002	0.182	0.168	-0.140
	p-value	**0.014**	0.102	0.987	0.102	0.131	0.208
Nasal LIPCOF	r	1.000	**0.351**	-0.049	-0.051	-0.079	**0.234**
	p-value		**0.001**	0.642	0.627	0.449	**0.024**
Temporal LIPCOF	r	**0.351**	1.000	0.110	-0.080	-0.084	0.096
	p-value	**0.001**		0.293	0.446	0.425	0.362
TBUT	r	-0.049	0.110	1.000	**-0.248**	0.091	0.058
	p-value	0.642	0.293		**0.016**	0.388	0.579
Corneal staining	r	-0.051	-0.080	**-0.248**	1.000	0.178	-0.116
	p-value	0.627	0.446	**0.016**		0.088	0.267
Obstructed MG	r	-0.079	-0.084	0.091	0.178	1.000	0.050
	p-value	0.449	0.425	0.388	0.088		0.632
Schirmer	r	**0.234**	0.096	0.058	-0.116	0.050	1.000
	p-value	**0.024**	0.362	0.579	0.267	0.632	
Mechanical sensitivity	r	0.065	0.168	0.109	-0.160	-0.038	-0.032
	p-value	0.535	0.110	0.299	0.129	0.717	0.765
Cold sensitivity	r	-0.136	-0.069	-0.200	**-0.231**	0.046	-0.110
	p-value	0.199	0.516	0.058	**0.027**	0.663	0.301
Hot sensitivity	r	-0.045	-0.024	-0.001	**0.223**	0.110	0.100
	p-value	0.675	0.823	0.993	**0.033**	0.301	0.347
Tactile sensitivity without anesthesia	r	0.153	0.166	-0.032	**-0.353**	**-0.349**	**0.257**
	p-value	0.142	0.112	0.762	**0.001**	**0.001**	**0.013**
Anesthetic challenge test	r	0.020	0.067	0.166	0.006	0.115	-0.073
	p-value	0.847	0.526	0.112	0.954	0.274	0.487

Tactile sensitivity with anesthesia	r	-0.169	0.163	0.077	-0.066	-0.045	0.112
	p-value	0.105	0.119	0.462	0.528	0.671	0.287
Nerve number	r	0.136	0.022	0.116	**-0.281**	**-0.255**	0.075
	p-value	0.198	0.833	0.271	**0.007**	**0.014**	0.476
Nerve density	r	0.128	0.015	0.103	**-0.236**	**-0.288**	0.003
	p-value	0.222	0.887	0.328	**0.023**	**0.005**	0.981
Nerve length	r	0.007	0.133	-0.018	-0.063	-0.104	-0.116
	p-value	0.946	0.207	0.866	0.551	0.323	0.271
Nerve reflectivity	r	-0.055	0.051	**-0.246**	0.011	-0.027	-0.080
	p-value	0.603	0.627	**0.018**	0.919	0.795	0.449
Microneuromas	r	**0.272**	0.109	-0.048	**0.246**	**0.220**	0.179
	p-value	**0.009**	0.299	0.651	**0.018**	**0.035**	0.088
Dendritic cells	r	-0.150	0.008	**-0.250**	**0.496**	0.033	-0.031
	p-value	0.152	0.937	**0.016**	**0.000**	0.754	0.769
Nerve branches	r	0.147	0.042	0.058	**-0.291**	**-0.270**	0.137
	p-value	0.163	0.689	0.585	**0.005**	**0.009**	0.193
Nerve tortuosity	r	0.093	**0.231**	-0.184	-0.053	-0.192	0.116
	p-value	0.380	**0.027**	0.080	0.615	0.067	0.272

P-values ≤ 0.05 were considered statistically significant and are shown in bold. LIPCOF: lid parallel conjunctival folds; MG: Meibomian glands; m-SIDEQ: modified Single-Item score Dry Eye Questionnaire; NRS: Numerical Rating Scale; OSDI: Ocular Surface Disease Index; r: correlation coefficient; TBUT: tear break-up time; total HOA RMS: root mean square of the total higher-order aberrations (from third to sixth order); VA: visual acuity; WBFPRS: Wong-Baker FACES® Pain Rating Scale.

Table S8. Correlations with corneal sensitivity parameters.

Variables		Mechanical sensitivity	Cold sensitivity	Hot sensitivity	Tactile sensitivity without anesthesia	Anesthetic challenge	Tactile sensitivity with anesthesia
OSDI	r	**-0.216**	**-0.242**	**0.255**	-0.198	**0.229**	0.092
	p-value	**0.039**	**0.021**	**0.015**	0.057	**0.027**	0.378
NRS	r	-0.184	-0.114	0.154	-0.115	0.160	0.045
	p-value	0.079	0.281	0.145	0.271	0.125	0.670
WBFPRS	r	-0.184	-0.114	0.154	-0.115	0.160	0.045
	p-value	0.079	0.281	0.145	0.271	0.125	0.670
m-SIDEQ	r	**-0.265**	**-0.304**	**0.307**	-0.180	**0.256**	0.085
	p-value	**0.011**	**0.003**	**0.003**	0.084	**0.013**	0.419
Anxiety	r	0.099	-0.023	0.113	-0.139	0.085	0.029
	p-value	0.350	0.831	0.285	0.185	0.418	0.782
Depression	r	0.038	-0.130	0.189	-0.132	0.068	0.070
	p-value	0.720	0.221	0.073	0.206	0.515	0.503
High contrast VA	r	**0.247**	-0.159	0.014	-0.094	0.100	**0.302**
	p-value	**0.019**	0.136	0.894	0.377	0.344	**0.004**
Low contrast VA	r	0.122	-0.143	0.038	-0.082	0.015	**0.287**
	p-value	0.262	0.190	0.729	0.448	0.888	**0.007**
Total HOA RMS	r	0.060	-0.104	0.097	-0.099	-0.022	**0.278**
	p-value	0.577	0.331	0.365	0.352	0.838	**0.008**
Meniscus area	r	0.079	0.042	-0.093	0.184	-0.073	0.165
	p-value	0.482	0.706	0.410	0.098	0.515	0.139
Meniscus depth	r	0.139	0.017	-0.070	0.158	-0.071	0.129
	p-value	0.216	0.880	0.533	0.156	0.524	0.247
Meniscus height	r	0.083	0.030	-0.101	0.175	-0.044	0.168
	p-value	0.461	0.793	0.369	0.117	0.697	0.131
Meniscus angle	r	-0.076	0.122	-0.053	-0.122	-0.087	-0.136
	p-value	0.501	0.277	0.635	0.273	0.439	0.223
Nasal LIPCOF	r	0.065	-0.136	-0.045	0.153	0.020	-0.169
	p-value	0.535	0.199	0.675	0.142	0.847	0.105
Temporal LIPCOF	r	0.168	-0.069	-0.024	0.166	0.067	0.163
	p-value	0.110	0.516	0.823	0.112	0.526	0.119
TBUT	r	0.109	-0.200	-0.001	-0.032	0.166	0.077
	p-value	0.299	0.058	0.993	0.762	0.112	0.462
Corneal staining	r	-0.160	**-0.231**	**0.223**	**-0.353**	0.006	-0.066
	p-value	0.129	**0.027**	**0.033**	**0.001**	0.954	0.528
Obstructed MG	r	-0.038	0.046	0.110	**-0.349**	0.115	-0.045
	p-value	0.717	0.663	0.301	**0.001**	0.274	0.671
Schirmer	r	-0.032	-0.110	0.100	**0.257**	-0.073	0.112
	p-value	0.765	0.301	0.347	**0.013**	0.487	0.287
Mechanical sensitivity	r	1.000	0.158	**-0.374**	0.002	0.025	-0.110
	p-value		0.135	**0.000**	0.985	0.815	0.296
Cold sensitivity	r	0.158	1.000	**-0.484**	0.174	-0.015	0.005
	p-value	0.135		**0.000**	0.098	0.889	0.965
Hot sensitivity	r	**-0.374**	**-0.484**	1.000	-0.119	-0.053	0.061
	p-value	**0.000**	**0.000**		0.261	0.617	0.564
Tactile sensitivity without anesthesia	r	0.002	0.174	-0.119	1.000	-0.112	0.146
	p-value	0.985	0.098	0.261		0.286	0.161

Anesthetic challenge test	r	0.025	-0.015	-0.053	-0.112	1.000	-0.092
	p-value	0.815	0.889	0.617	0.286		0.379
Tactile sensitivity with anesthesia	r	-0.110	0.005	0.061	0.146	-0.092	1.000
	p-value	0.296	0.965	0.564	0.161	0.379	
Nerve number	r	0.000	0.012	-0.008	**0.328**	-0.165	0.018
	p-value	0.998	0.912	0.940	**0.001**	0.115	0.867
Nerve density	r	0.038	0.020	-0.068	**0.313**	**-0.215**	-0.063
	p-value	0.719	0.849	0.523	**0.002**	**0.040**	0.548
Nerve length	r	0.115	0.174	**-0.230**	0.177	0.008	-0.012
	p-value	0.276	0.102	**0.029**	0.091	0.940	0.909
Nerve reflectivity	r	0.103	0.066	-0.017	0.098	0.030	-0.026
	p-value	0.333	0.539	0.873	0.355	0.780	0.808
Microneuromas	r	-0.061	-0.106	**0.207**	-0.062	0.034	-0.109
	p-value	0.567	0.318	**0.050**	0.558	0.744	0.301
Dendritic cells	r	-0.114	-0.044	0.046	-0.056	0.182	-0.042
	p-value	0.283	0.678	0.667	0.597	0.083	0.688
Nerve branches	r	-0.007	0.052	-0.021	**0.392**	-0.174	0.037
	p-value	0.946	0.624	0.845	**0.000**	0.097	0.727
Nerve tortuosity	r	-0.002	0.019	-0.047	**0.242**	0.014	0.054
	p-value	0.986	0.862	0.661	**0.020**	0.892	0.609

P-values ≤ 0.05 were considered statistically significant and are shown in bold. LIPCOF: lid parallel conjunctival folds; MG: Meibomian glands; m-SIDEQ: modified Single-Item score Dry Eye Questionnaire; NRS: Numerical Rating Scale; OSDI: Ocular Surface Disease Index; r: correlation coefficient; TBUT: tear break-up time; total HOA RMS: root mean square of the total higher-order aberrations (from third to sixth order); VA: visual acuity; WBFPRS: Wong-Baker FACES® Pain Rating Scale.

Table S9. Correlations with subbasal corneal nerve plexus parameters.

Variables		Nerve number	Nerve density	Nerve length	Nerve reflectivity	Micro-neuromas	Dendritic cells	Nerve branches	Nerve tortuosity
OSDI	r	**-0.257**	**-0.249**	-0.009	0.034	**0.219**	**0.429**	-0.187	0.153
	p-value	**0.013**	**0.017**	0.934	0.745	**0.036**	**0.000**	0.074	0.146
NRS	r	**-0.337**	**-0.369**	-0.061	-0.055	**0.405**	0.132	**-0.261**	0.083
	p-value	**0.001**	**0.000**	0.564	0.606	**0.000**	0.210	**0.012**	0.430
WBFPRS	r	**-0.291**	**-0.335**	-0.099	-0.125	**0.416**	0.130	**-0.240**	0.048
	p-value	**0.005**	**0.001**	0.345	0.237	**0.000**	0.215	**0.021**	0.648
m-SIDEQ	r	**-0.301**	**-0.275**	0.035	-0.040	**0.293**	**0.440**	**-0.252**	0.107
	p-value	**0.004**	**0.008**	0.737	0.708	**0.005**	**0.000**	**0.015**	0.310
Anxiety	r	-0.004	0.091	0.113	0.138	**0.253**	0.099	-0.037	0.182
	p-value	0.971	0.387	0.283	0.191	**0.015**	0.347	0.725	0.083
Depression	r	-0.019	0.048	0.085	0.091	0.150	0.115	0.011	0.164
	p-value	0.854	0.649	0.418	0.387	0.154	0.276	0.915	0.119
High contrast VA	r	-0.076	-0.031	0.096	0.181	0.100	-0.121	-0.084	0.098
	p-value	0.476	0.773	0.367	0.088	0.348	0.257	0.434	0.358
Low contrast VA	r	-0.040	-0.048	-0.014	0.172	0.101	-0.109	-0.047	0.124
	p-value	0.715	0.658	0.894	0.112	0.354	0.314	0.666	0.251
Total HOA RMS	r	-0.073	-0.169	-0.034	0.135	-0.012	0.037	-0.062	0.056
	p-value	0.494	0.112	0.750	0.203	0.909	0.732	0.561	0.602
Meniscus area	r	**0.254**	**0.241**	0.075	-0.206	-0.099	-0.190	**0.274**	0.196
	p-value	**0.022**	**0.030**	0.506	0.065	0.381	0.089	**0.013**	0.079
Meniscus depth	r	**0.274**	**0.264**	0.070	-0.159	-0.120	-0.216	**0.296**	0.189
	p-value	**0.013**	**0.017**	0.533	0.156	0.288	0.053	**0.007**	0.091
Meniscus height	r	**0.228**	**0.225**	0.086	-0.186	-0.069	-0.141	**0.242**	0.207
	p-value	**0.041**	**0.044**	0.446	0.096	0.541	0.210	**0.030**	0.063
Meniscus angle	r	-0.140	-0.104	-0.030	-0.036	0.013	0.074	-0.112	-0.194
	p-value	0.213	0.353	0.790	0.748	0.910	0.514	0.320	0.082
Nasal LIPCOF	r	0.136	0.128	0.007	-0.055	**0.272**	-0.150	0.147	0.093
	p-value	0.198	0.222	0.946	0.603	**0.009**	0.152	0.163	0.380
Temporal LIPCOF	r	0.022	0.015	0.133	0.051	0.109	0.008	0.042	**0.231**
	p-value	0.833	0.887	0.207	0.627	0.299	0.937	0.689	**0.027**
TBUT	r	0.116	0.103	-0.018	**-0.246**	-0.048	**-0.250**	0.058	-0.184
	p-value	0.271	0.328	0.866	**0.018**	0.651	**0.016**	0.585	0.080
Corneal staining	r	**-0.281**	**-0.236**	-0.063	0.011	**0.246**	**0.496**	**-0.291**	-0.053
	p-value	**0.007**	**0.023**	0.551	0.919	**0.018**	**0.000**	**0.005**	0.615
Obstructed MG	r	**-0.255**	**-0.288**	-0.104	-0.027	**0.220**	0.033	**-0.270**	-0.192
	p-value	**0.014**	**0.005**	0.323	0.795	**0.035**	0.754	**0.009**	0.067
Schirmer	r	0.075	0.003	-0.116	-0.080	0.179	-0.031	0.137	0.116
	p-value	0.476	0.981	0.271	0.449	0.088	0.769	0.193	0.272
Mechanical sensitivity	r	0.000	0.038	0.115	0.103	-0.061	-0.114	-0.007	-0.002
	p-value	0.998	0.719	0.276	0.333	0.567	0.283	0.946	0.986
Cold sensitivity	r	0.012	0.020	0.174	0.066	-0.106	-0.044	0.052	0.019
	p-value	0.912	0.849	0.102	0.539	0.318	0.678	0.624	0.862
Hot sensitivity	r	-0.008	-0.068	**-0.230**	-0.017	**0.207**	0.046	-0.021	-0.047
	p-value	0.940	0.523	**0.029**	0.873	**0.050**	0.667	0.845	0.661
Tactile sensitivity without anesthesia	r	**0.328**	**0.313**	0.177	0.098	-0.062	-0.056	**0.392**	**0.242**
	p-value	**0.001**	**0.002**	0.091	0.355	0.558	0.597	**0.000**	**0.020**
	r	-0.165	**-0.215**	0.008	0.030	0.034	0.182	-0.174	0.014

Anesthetic challenge test	p-value	0.115	**0.040**	0.940	0.780	0.744	0.083	0.097	0.892
Tactile sensitivity with anesthesia	r	0.018	-0.063	-0.012	-0.026	-0.109	-0.042	0.037	0.054
	p-value	0.867	0.548	0.909	0.808	0.301	0.688	0.727	0.609
Nerve number	r	1.000	**0.916**	-0.133	-0.152	**-0.223**	**-0.284**	**0.912**	**0.405**
	p-value		**0.000**	0.205	0.147	**0.032**	**0.006**	**0.000**	**0.000**
Nerve density	r	**0.916**	1.000	0.163	-0.076	**-0.283**	**-0.253**	**0.822**	**0.342**
	p-value	**0.000**		0.121	0.470	**0.006**	**0.015**	**0.000**	**0.001**
Nerve length	r	-0.133	0.163	1.000	**0.261**	**-0.254**	-0.089	-0.116	-0.051
	p-value	0.205	0.121		**0.012**	**0.015**	0.400	0.271	0.631
Nerve reflectivity	r	-0.152	-0.076	**0.261**	1.000	0.038	-0.029	-0.135	0.056
	p-value	0.147	0.470	**0.012**		0.717	0.785	0.201	0.596
Microneuromas	r	**-0.223**	**-0.283**	**-0.254**	0.038	1.000	0.003	**-0.214**	-0.007
	p-value	**0.032**	**0.006**	**0.015**	0.717		0.980	**0.040**	0.946
Dendritic cells	r	**-0.284**	**-0.253**	-0.089	-0.029	0.003	1.000	**-0.255**	0.097
	p-value	**0.006**	**0.015**	0.400	0.785	0.980		**0.014**	0.359
Nerve branches	r	**0.912**	**0.822**	-0.116	-0.135	**-0.214**	**-0.255**	1.000	**0.523**
	p-value	**0.000**	**0.000**	0.271	0.201	**0.040**	**0.014**		**0.000**
Nerve tortuosity	r	**0.405**	**0.342**	-0.051	0.056	-0.007	0.097	**0.523**	1.000
	p-value	**0.000**	**0.001**	0.631	0.596	0.946	0.359	**0.000**	

P-values ≤ 0.05 were considered statistically significant and are shown in bold. LIPCOF: lid parallel conjunctival folds; MG: Meibomian glands; m-SIDEQ: modified Single-Item score Dry Eye Questionnaire; NRS: Numerical Rating Scale; OSDI: Ocular Surface Disease Index; r: correlation coefficient; TBUT: tear break-up time; total HOA RMS: root mean square of the total higher-order aberrations (from third to sixth order); VA: visual acuity; WBFPRS: Wong-Baker FACES® Pain Rating Scale.
